# Risk Factors for Pathologic Fracture in Dogs With Confirmed or Suspected Appendicular Osteosarcoma Treated With Low‐ or Standard‐Dose‐Rate Palliative External Beam Radiation Therapy

**DOI:** 10.1111/vru.70245

**Published:** 2026-09-28

**Authors:** Andrew Kurtzman, Elizabeth Rappleyea, Matt Stewart, Jayme Looper, Audrey Billhymer, Kim A. Selting

**Affiliations:** ^1^ Department of Veterinary Clinical Medicine University of Illinois Urbana Illinois USA; ^2^ Department of Veterinary Clinical Sciences Louisiana State University Baton Rouge Louisiana USA

**Keywords:** canine, dose‐rate, osteosarcoma, pathologic fracture, radiation

## Abstract

Pathologic fracture is a complication of bone cancer with probable increased risk following radiation therapy (RT). Low‐dose‐rate (LDR) RT can spare late‐responding normal tissues by allowing intrafraction DNA repair, which could also impact tumor response. This retrospective cohort study evaluated efficacy and fracture rate in canine patients (*n* = 184) with confirmed or suspected appendicular osteosarcoma that received 10 Gy × 2 palliative RT on consecutive days with LDR (cobalt‐60) or standard‐dose‐rate (SDR, linear accelerator). The overall fracture rate was 21.7%. Patients treated with SDR (600 cGy/min, *n* = 46) and LDR (range 21.9–83.29 cGy/min, *n* = 152) did not differ in pathologic fracture rates (22.4% vs. 17.4%, respectively, *p* = 0.54) or median survival times (231 vs. 248.5 days, respectively, *p* = 0.448). Pathologic fracture rates did not differ on the basis of weight (<35, 35–50, and >50 kg) or location (24% forelegs, 18% hindlegs, *p* = 0.44). However, no dog (*n* = 7) weighing less than 20 kg developed a fracture. Radiographs prior to RT were subjectively assessed for bone integrity. Dogs with predominantly lytic lesions did not have a significantly higher pathologic fracture rate (21.2%–31.69% lytic/mixed vs. 10%–12.5% proliferative, *p* = 0.054), suggesting that pretreatment radiographic appearance does not reflect fracture risk. Additionally, the presence of a pretreatment cortical bone defect was not associated with eventual fracture (*p* = 0.7). Adjunctive chemotherapy improved survival time significantly (*p* = 0.01). On the basis of our data, it is reasonable to compare response rates and survival outcomes in published studies using cobalt irradiation with reports using linear accelerators.

## 1 Introduction

Osteosarcoma (OSA) comprises up to 85% of malignant primary bone tumors in dogs. OSA primarily affects middle‐aged and older large and giant breed dogs (median age 7 years), although a bimodal age distribution exists with a smaller, earlier peak at age 18–24 months. The majority (75%) of OSA occur in the appendicular skeleton with predilection for metaphyses, front limbs over rear limbs, and specific sites (distal radius, proximal humerus, distal femur, distal tibia, and proximal tibia). Presenting complaints may include lameness (weight‐bearing or non‐weight‐bearing, most often related to pain), swelling over the affected site, pain, or recent trauma. Radiographically, OSA bone lesions typically show cortical lysis, reactive periosteum, and associated soft tissue swelling and do not cross the joint space [1]. Cytology with alkaline phosphatase (ALP) staining supports a presumptive diagnosis of OSA, though definitive diagnosis requires a bone core biopsy and histopathology [2]. Metastasis typically occurs early in disease development. Thoracic radiographs or CT scans are included in staging work‐ups to evaluate pulmonary metastasis status, although only 15% will have visible metastasis at diagnosis [3].

Standard of care currently involves amputation of the affected limb for local control and pain relief, followed by single‐agent or combination chemotherapy protocols to delay metastasis [1]. Some dogs are not good candidates for amputation due to concurrent musculoskeletal or neurologic pathology or owner refusal. In these cases, radiation therapy (RT) may be pursued. The primary quality of life concern with OSA tends to be pain, and thus, many protocols exist for palliative local care [4, 5]. Humans with bone neoplasia describe both ongoing and breakthrough pain that is believed to be present in dogs as well [1, 6, 7]. This bone pain is likely multifactorial and proposed to originate from osteoclast‐mediated bone resorption and associated microfractures, inflammatory cell activation, neuropathic processes, and secretion of nociceptive ligands [8, 9]. Radiotherapy reduces bone pain through several proposed mechanisms: interference with inflammatory cells and mediators, disruption of osteolysis by osteoclasts, and reduced gross tumor size [6, 7].

Multiple studies have evaluated the use of two to four high‐dose per fraction (6–10 Gy per fraction) protocols, resulting in 75%–90% of dogs responding favorably and subjectively appearing less painful [1, 5, 6, 10, 11, 12, 13, 14, 15]. Factors that can vary with radiation and potentially affect outcome include dose per fraction, total dose, interval between fractions, and dose rate. A decreased dose rate (with an increased treatment time) could allow improved DNA repair in irradiated tumor cells [16]. Nonhomologous end‐joining (NHEJ) and homologous recombination (HR) contribute to the fast and slow components of double‐strand DNA break repair with half‐lives in the range of 5–30 min and 2–5 h, respectively [17]. It is reasonable that the difference between 2 min (linear accelerator) and 40 min (cobalt‐60, or Co‐60) to deliver 10 Gy could result in differences in NHEJ, which is more error‐prone, without appreciable differences in HR.

The University of Illinois used a Co‐60 source for palliative OSA radiotherapy until 2019. Co‐60 decays by emitting two gamma rays with an average energy of 1.25 MV. As the half‐life of Co‐60 is approximately 5.2 years, natural decay leading to decreased dose rate occurred steadily during the gamma emitter's usage [18]. From mid 2019, dogs were treated with a linear accelerator at a standard‐dose rate (SDR) of 600 cGy/min. Tumors with low *α*/*β* ratios (such as osteosarcoma cell lines), as with late‐responding tissues, including nerves and blood vessels, might be more affected by low‐dose rates (LDRs), as early responding tissues are less susceptible to the dose‐rate effect than late‐responding tissues [16, 17, 19]. The primary aim of this retrospective cohort study was to assess a single protocol (10 Gy × 2) for palliative irradiation of canine appendicular osteosarcoma and determine whether there were any differences in efficacy or toxicity over time, as a function of dose rate. We also evaluated other potential risk factors related to rates of pathologic fracture such as weight, tumor location, and radiographic appearance. We hypothesized that high (600 Gy/min) radiation dose rate, limb loading (body weight and forelimb vs. hind limb), and osteolytic status of the lesions, as assessed from radiographs, are risk factors for pathological fracture in canine appendicular OSA patients [20].

## 2 Materials and Methods

### 2.1 Subjects

A review of medical records for documented cases receiving RT from April 2009 to July 2024 at the University of Illinois was performed. Inclusion criteria were pet dogs with a suspected or confirmed diagnosis of OSA on the basis of radiographs, cytology with ALP staining, or histopathology, and who received a 10 Gy × 2 on consecutive days radiation treatment plan, and an available radiation delivery dose‐rate. Exclusion criteria were patients with a non‐appendicular skeletal mass location or patients that failed to complete the planned RT protocol.

### 2.2 Data Collection

Data were collected from the medical records at the University of Illinois, referring veterinary medical records, and the radiation delivery planning system (Varian Eclipse v15). Variables assessed included specific anatomic location, sex and neuter status, breed, date of birth, date of diagnosis, age and weight at diagnosis, date and nature of first progressive event, calculated time to progression (TTP), the presence of pathologic fracture, primary cause of death or date of last known follow‐up, date of death, whether cytology/histopathology/chest radiographs were performed, radiation dose rate (cGy/min), use of chemotherapy, use of nonsteroidal anti‐inflammatory drugs (NSAIDs) before and after radiation, use of opioids before and after radiation, and the use of other pain medications before and after radiation. Patients were divided by weight categories of <35, 35–50, or >50 kg to approximate small/medium breed dogs, large breed dogs, and giant breed dogs, respectively, as well as being natural breakpoints in the data set that led to a relatively similar distribution of cases among the groups.

Dogs that developed pathologic fractures were identified when exhibiting a sudden worsening in pain and non‐weight‐bearing lameness, accompanied by sudden external change in the appearance of the tumor site. Radiographic confirmation was available in 37 cases, defined by the appearance of a new radiolucent fracture line accompanied in some cases by malalignment. Three dogs were classified as having a suspected pathologic fracture without radiographic confirmation, on the basis of the clinical changes noted above. Dogs reported to have progressive pain without such clinical signs were considered to have progressive disease and were not classified as having pathologic fracture. Dogs were classified as having a suspected pathologic fracture without radiographic confirmation if the record reflected a sudden worsening in pain and non‐weight‐bearing lameness, especially when accompanied by sudden change in the appearance of the tumor area such as swelling. Dogs that were reported to have progressive pain without such clinical signs were considered to have progressive disease and were not classified as having a pathologic fracture. Pathologic fractures were divided into events occurring < or > 3 months after RT to separate patients with pathologic fracture due to the OSA lesion itself from those occurring as a late effect of RT. TTP was determined by the first event after RT (tumor growth, pathologic fracture, or metastasis). Eleven patients had a first event (such as sepsis or IMHA) that was not related to their diagnosis of OSA, which was considered an endpoint as they often limited life. For the purpose of this study, all‐cause mortality without censoring was used. Neuter status in relation to other variables was not statistically evaluated due to the low number of intact patients in either group. We intended to evaluate the possible effect of bisphosphonate use on fracture risk; however, almost all dogs (97.3%, *n* = 179) received adjunctive IV bisphosphonate therapy, precluding any comparisons.

Pre‐treatment radiographic images from 161 cases were reviewed by three of the authors (AK and KAS [ACVR boarded radiation oncologist] consensus and AB [ACVR boarded radiologist] independently). Radiographic images of the lesions were subjectively classified as either “predominantly lytic,” “predominantly proliferative,” or “mixed lytic/proliferative.” Images were also evaluated for cortical bone loss of at least one cortex.

### 2.3 Treatment Protocol

#### 2.3.1 Radiation Therapy

All patients were treated with external beam radiation using either Co‐60 (Theratron 780, MDS Nordion Inc./Best Theratronics, Canada) or 6 MV photons delivered via linear accelerator (Varian TrueBeam, Palo Alto, CA). Most dogs were positioned using tape stabilization of the limb, and some were positioned using a vacuum bag immobilization device (Vac‐Lok), based on tumor location and clinician preference. The dose prescription was palliative intent, 10 Gy × treatments given on consecutive days, with a dose rate of delivery being either “low‐dose‐rate” ranging between 21.9 and 83.29 cGy/min using Co‐60, or a “standard‐dose rate” of 600 cGy/min using the linear accelerator. Treatments were delivered via manual setup to the isocenter of the target (calculated to the central axis), using source‐to‐axis (SAD) dosing, with two, equally weighted, parallel‐opposed fields. Bolus was not used in most cases, given the shallow depth of maximum dose (*D*_max_) of 5 mm with Co‐60, and the goal of minimizing the risk of skin effects in all cases because the intent of treatment was palliative. In some cases, a CT‐guided computer‐based plan was used for proximal humerus (*n* = 10) or scapular (*n* = 3) lesions. Annual quality assurance was performed for the Co‐60 machine, and daily, monthly, and annual physics checks were performed for the linear accelerator.

#### 2.3.2 Chemotherapy

Of the patients that received chemotherapy, the majority received platinum‐based chemotherapy protocols. However, these were not standardized among patients and were not evaluated by treatment subtype. Other protocols included toceranib phosphate (Palladia) alone, Palladia and vinblastine, doxorubicin alone, doxorubicin and cyclophosphamide, cyclophosphamide alone, cyclophosphamide and Palladia, doxorubicin and PAC‐1, CCNU alone, or rapamycin alone. Patients were divided into groups that received or did not receive chemotherapy.

### 2.4 Statistical Evaluation

Statistical analyses were carried out using GraphPad Prism V 10.6.1. Data sets were assessed for normality with the Shapiro–Wilk normality test. Survival outcomes were plotted as Kaplan–Meier survival curves and analyzed by Mantel–Cox log‐rank tests and also by Gehan–Breslow–Wilcoxon tests, which account for a substantial number of deaths in the early stages of a study.

The influences of “radiation dose rate,” “chemotherapeutic administration,” “limb location,” and “cortical bone integrity” on pathological fracture incidence were assessed by Fisher's exact tests. The impact of “body weight” on pathological fracture incidence was assessed by Mann–Whitney tests. The chi‐square test was used to compare fracture incidences across radiographic categories.

For all statistical comparisons, significance was set at *p* < 0.05.

## 3 Results

One hundred and ninety‐six potential subjects were identified at the initial screening. Of these, 12 were excluded (three patients did not complete the RT protocol, three had a non‐OSA diagnosis [1 liposarcoma, 1 chondrosarcoma, 1 hemangiosarcoma], two had incomplete medical records with no follow‐up, two were negative ALP‐staining sarcomas, and two patients had non‐appendicular locations). Ultimately, 184 patients were selected for statistical analyses. In total, 198 individual radiation protocols were evaluated, with 12 patients having two separate radiation treatments and one patient having three separate radiation treatments. The median age at diagnosis was 8.88 years (range 1.95–14.55 years). Ninety‐nine treated dogs were neutered males, 91 were spayed females, seven were intact males, and one was an intact female. The most common breeds were 39 mixed breed dogs, 26 Labrador Retrievers, 17 Golden Retrievers, 13 Rottweilers, 13 St. Bernards, and 12 Great Danes, with the remainder being a mix of breeds (Table 1). The median weight at diagnosis was 42.48 kg (range 7.9–83.6 kg). The weight categories were <35 kg (*n* = 45 with seven dogs weighing less than 20 kg), between 35 and 50 kg (*n* = 81), and >50 kg (*n* = 58). Dogs treated more than once did not change enough in weight to change categories.

**TABLE 1:** Signalment.

Variable	All dose rates (*n* = 184)	Low‐dose rate (*n* = 139)	Standard‐dose rate (*n* = 45)
Age (years) (range)	Median 8.88 (1.95–14.58)	Median 8.89 (3.67–14.58)	Median 8.75 (1.95–14.55)
Weight (kg) (range)	Median 42.79 (7.9–83.6)	Median 42.68 (11–83.6)	Median 42.91 (7.9–69.55)
Patients <35 kg	45	32	13
>Patients 35–50 kg	81	61	20
Patients >50 kg	58	46	12
Female spayed	81	65	16
Female intact	1	1	0
Male neutered	95	67	28
Male intact	7	6	1
Mixed breed dog	39	29	10
Labrador Retriever	26	22	4
Golden Retriever	17	15	2
Rottweiler	13	11	2
St. Bernard	13	8	5
Great Dane	13	10	3
Great Pyrenees	7	4	3
Irish Wolfhound	7	5	2
Mastiff	6	6	0
Newfoundland	4	4	0
Greyhound	4	3	1
Old English Sheepdog	4	2	2
German Shepherd	4	3	1
Doberman	3	3	0
Alaskan Malamute	3	3	0
Bernese Mountain Dog	3	1	2
Pit Bull Terrier	2	2	0
Bouvier de Flandres	2	2	0
Leonberger	2	1	1
Various single purebred canines	12	5—Swiss Mountain Dog, Shiba Inu, Catahoula Hound, Irish Setter, Siberian Husky	7—Standard Poodle, Australian Shepherd, Chesapeake Bay Retriever, Rhodesian Ridgeback, Cane Corso, Flat Coated Retriever, Cairn Terrier
Radiation site (per treatment protocol, *n* = 198)		Low‐dose rate (per treatment protocol, *n* = 152)	Standard‐dose rate (per treatment protocol, *n* = 46)
Front limb	148	116	32
Hind limb	50	36	14

The overall rate of pathologic fracture was 21.7% and the median time to fracture was 111 days. Patients receiving SDR RT (*n* = 46) and LDR RT (*n* = 152) were not statistically different in pathologic fracture rates (22.4% vs. 17.4%, respectively, *p* = 0.54, Figure 1) or median survival times (231 vs. 248.5 days, respectively, *p* = 0.448; Figure 2).

**FIGURE 1:**
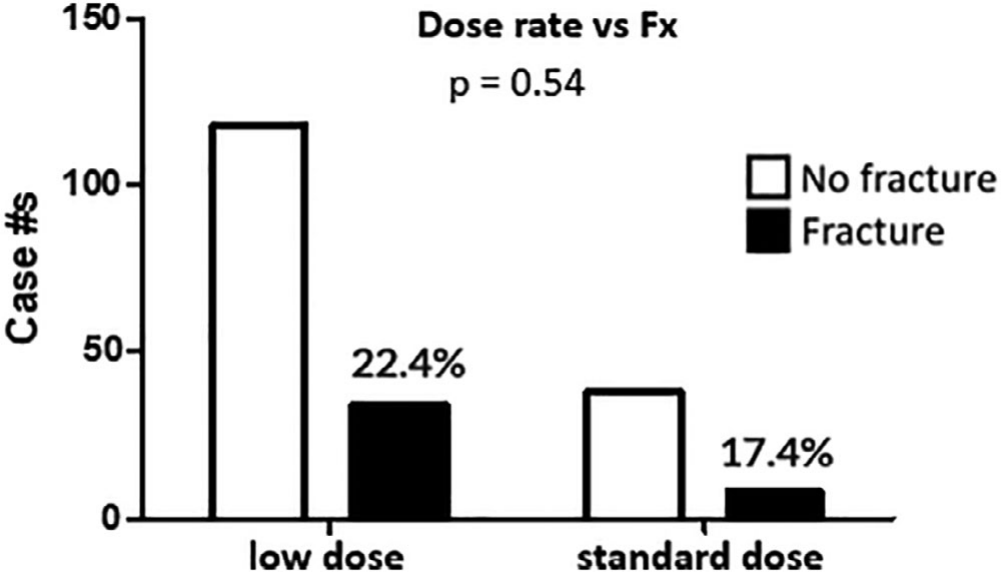
Comparison of low versus standard radiation dose rate of delivery versus incidence of pathologic fracture. Percentages listed per group represent fracture, no significant statistical differences (*p* = 0.54, Fisher's exact test).

**FIGURE 2:**
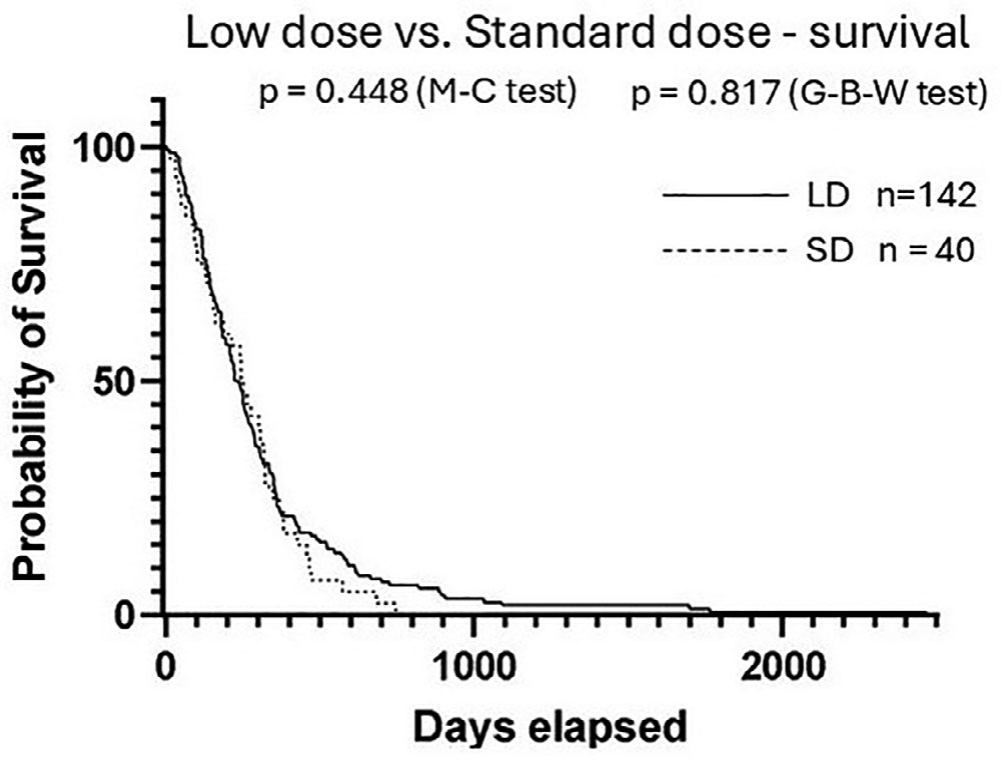
Comparison of low‐dose (LD) and standard dose (SD) rate radiation delivery versus overall survival time, no significant statistical differences (*p* = 0.448, Mantel–Cox test, and 0.817, Gehan–Breslow–Wilcoxon test).

There was no statistically significant difference in pathologic fracture rates between the three weight groups (20%, 21.18%, and 26.98%, respectively, Figure 3) or between forelimb and hind limb (front 24%, back 18%, *p* = 0.44; Figure 4). Of note, none of the seven dogs in the cohort weighing less than 20 kg developed a fracture (Figure 3). There were no statistical differences in median survival times for patients of various weight groups (<35, 35–50, and >50 kg, *p* = 0.624 and 0.358, Figure 5).

**FIGURE 3:**
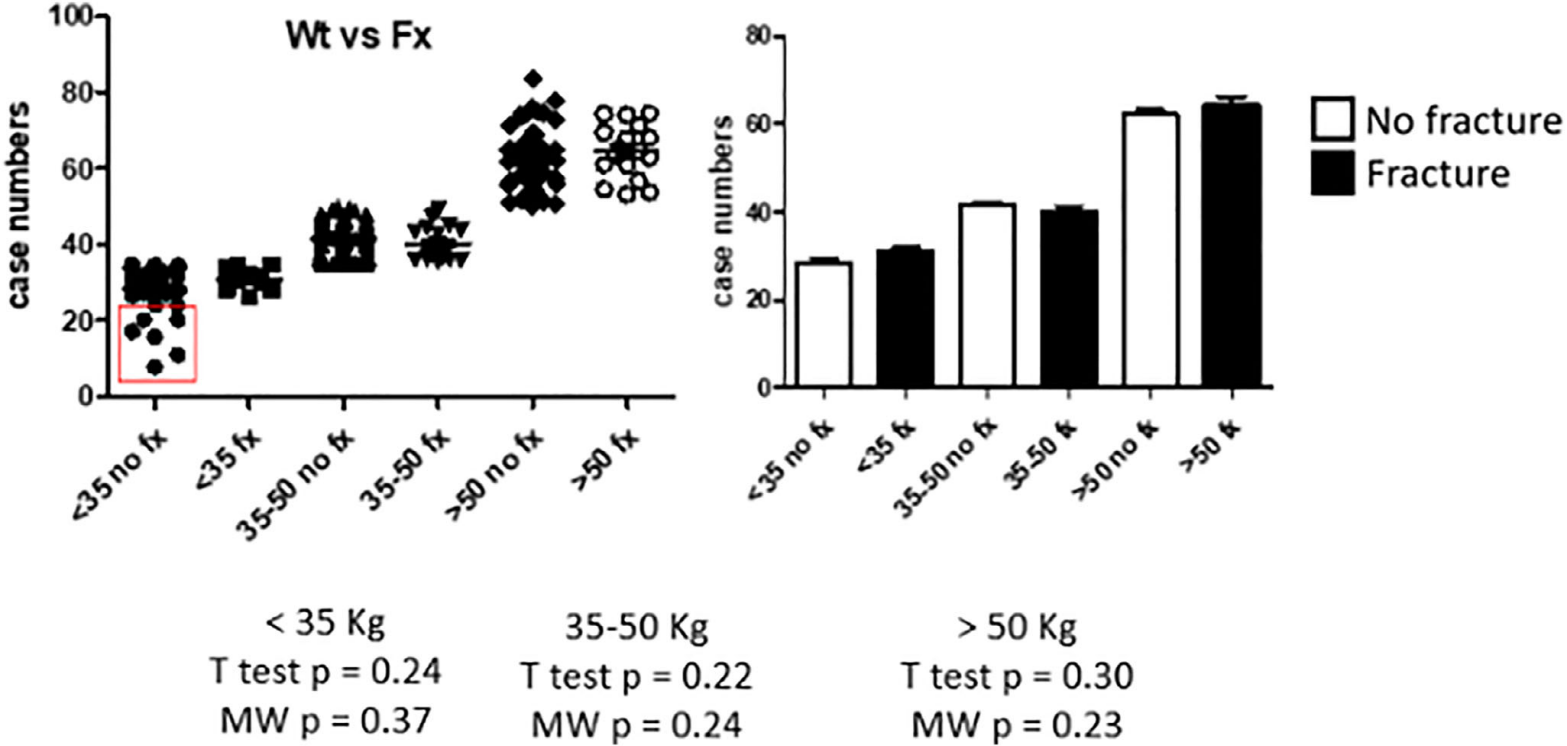
Comparison of different weight categories versus incidence of pathologic fracture, each symbol represents single treatment in group, red box highlighting patients less than 20 kg did not have any pathologic fractures, no significant statistical differences (<35 kg *p* = 0.24 and 0.37, 35–50 kg *p* = 0.22 and 0.24, >50 kg *p* = 0.30 and 0.23, respectively, for *t*‐test and Mann–Whitney test).

**FIGURE 4:**
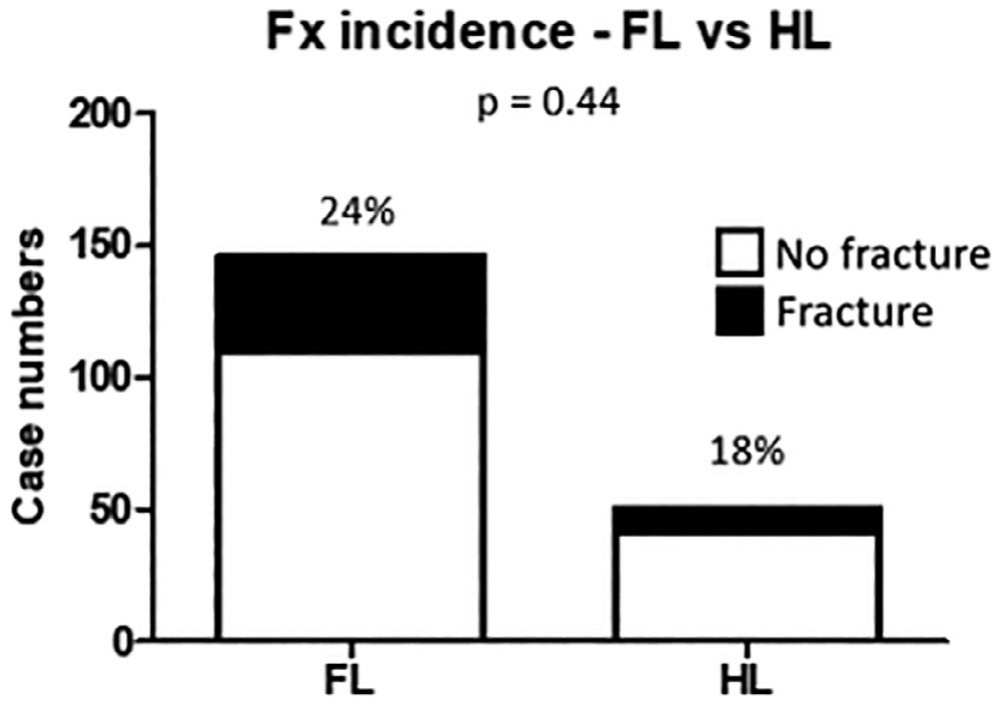
Comparison of forelimb (FL) or hind limb (HL) lesion location versus incidence of pathologic fracture. Percentages listed per group represent percentages fractured, no significant statistical differences (*p* = 0.44, Mann–Whitney test).

**FIGURE 5:**
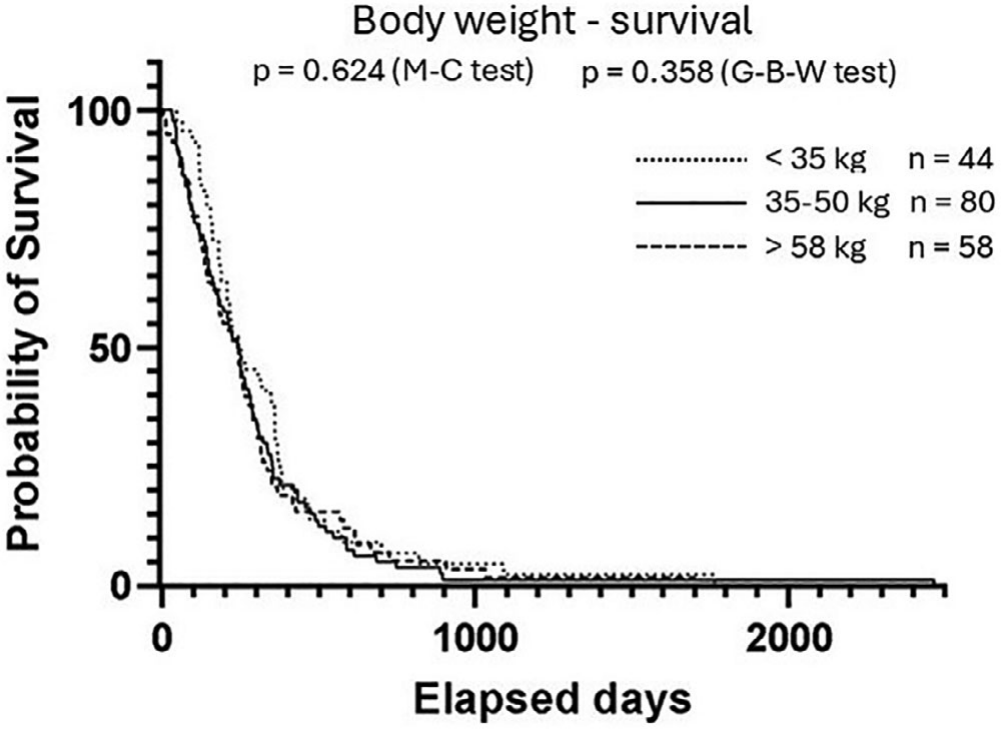
Comparison of patients with different weight categories versus survival time, no statistically significant differences (*p* = 0.624, Mantel–Cox test, and 0.358 Gehan–Breslow–Wilcoxon test).

Fifty‐five (27.8%) patients received adjunctive chemotherapy. Most of these patients (61.8%, *n* = 34) received a platinum‐based chemotherapy protocol. There was a statistically significant improvement in median survival times when comparing patients that received chemotherapy versus those that did not (297.5 vs. 213.5 days, respectively, *p* = 0.01, Figure 6).

**FIGURE 6:**
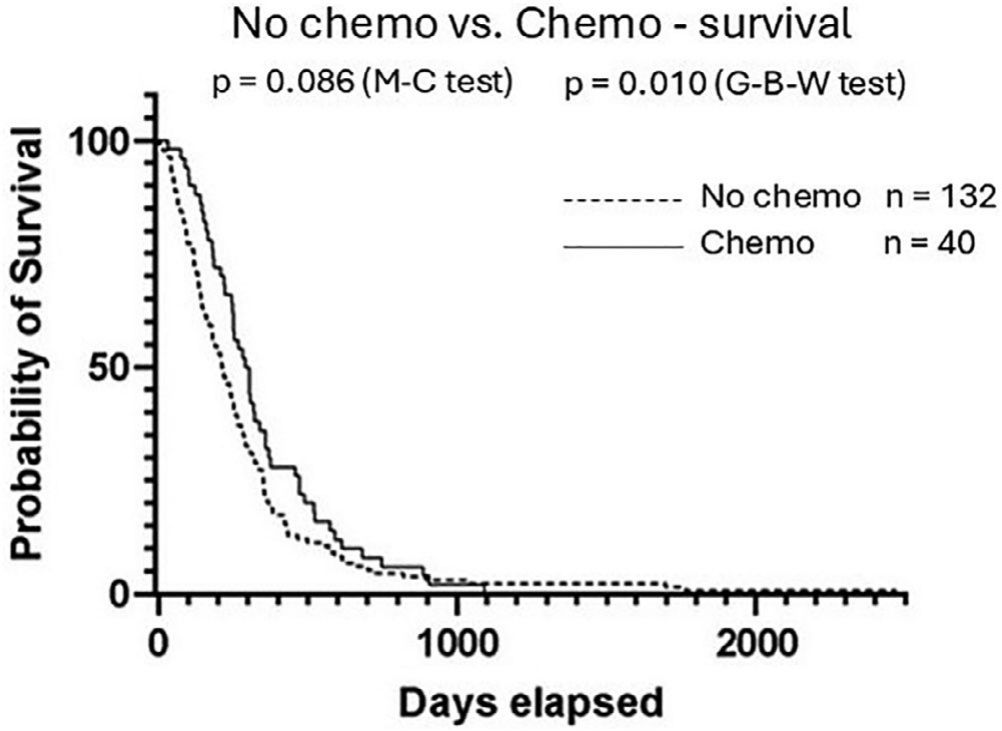
Comparison of patients with or without any chemotherapy use versus survival time in days, statistically significant difference (*p* = 0.086, Mantel–Cox test, and 0.010, Gehan–Breslow–Wilcoxon test).

The overall agreement rate between authors AK/KAS (Observer 1) and author AB (Observer 2) for the categorization of radiographic images was 67.65%. Considering the board‐certified radiologist (Observer 2) to be the authority, underestimation of the proliferative nature of the tumor accounted for 21.76% of cases and overestimation accounted for 10.59%. The overall agreement rate regarding cortical bone loss was 61.25%. Only two of 62 radiographic image sets overestimated cortical bony lysis by Observer 1 when Observer 2 did not agree, so overestimation and underestimation were grouped together as general disagreement.

The overall fracture rate of lesions considered primarily lytic, mixed lytic/proliferative, or primarily proliferative was 26.9%, 21.2%, and 10.0%, respectively, based on evaluation by Observer 1 and 21.6%, 32.7%, and 12.5%, respectively, based on evaluation by Observer 2. Dogs with “a predominantly proliferative” designation were less likely to have a pathologic fracture, though this did not reach statistical significance (Observer 1, *p* = 0.16; Observer 2, *p* = 0.054, Figures 7 and 8). The overall fracture rate for lesions with or without a pretreatment defect in at least one cortex was 21% and 23.7%, respectively (*p* = 0.702, Figures 9 and 10).

**FIGURE 7:**
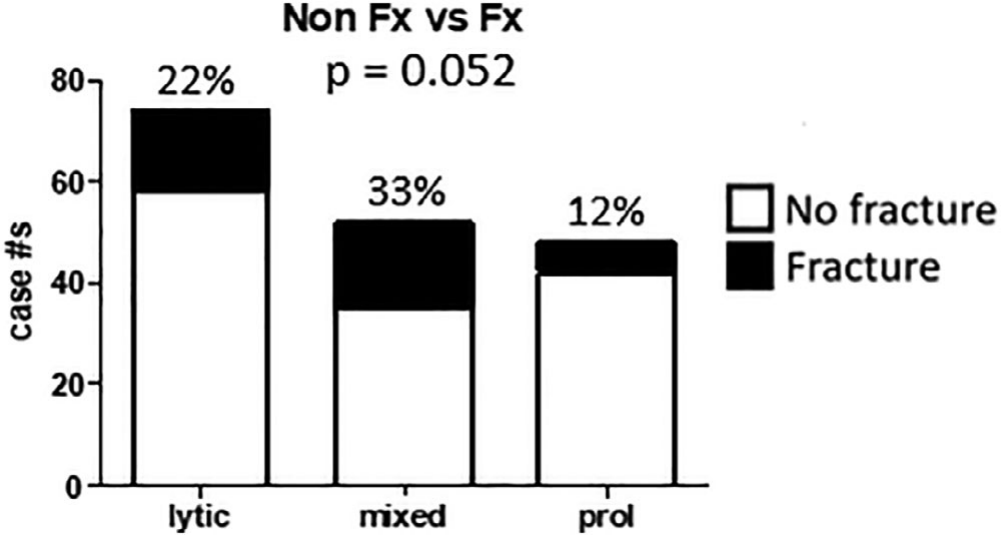
Comparison of lesion imaging characteristics versus incidence of pathologic fracture. These data are derived from the assessment of Observer 2. Percentages listed per group represent percentages fractured, no statistically significant differences (*p* = 0.054, chi‐square test).

**FIGURE 8:**
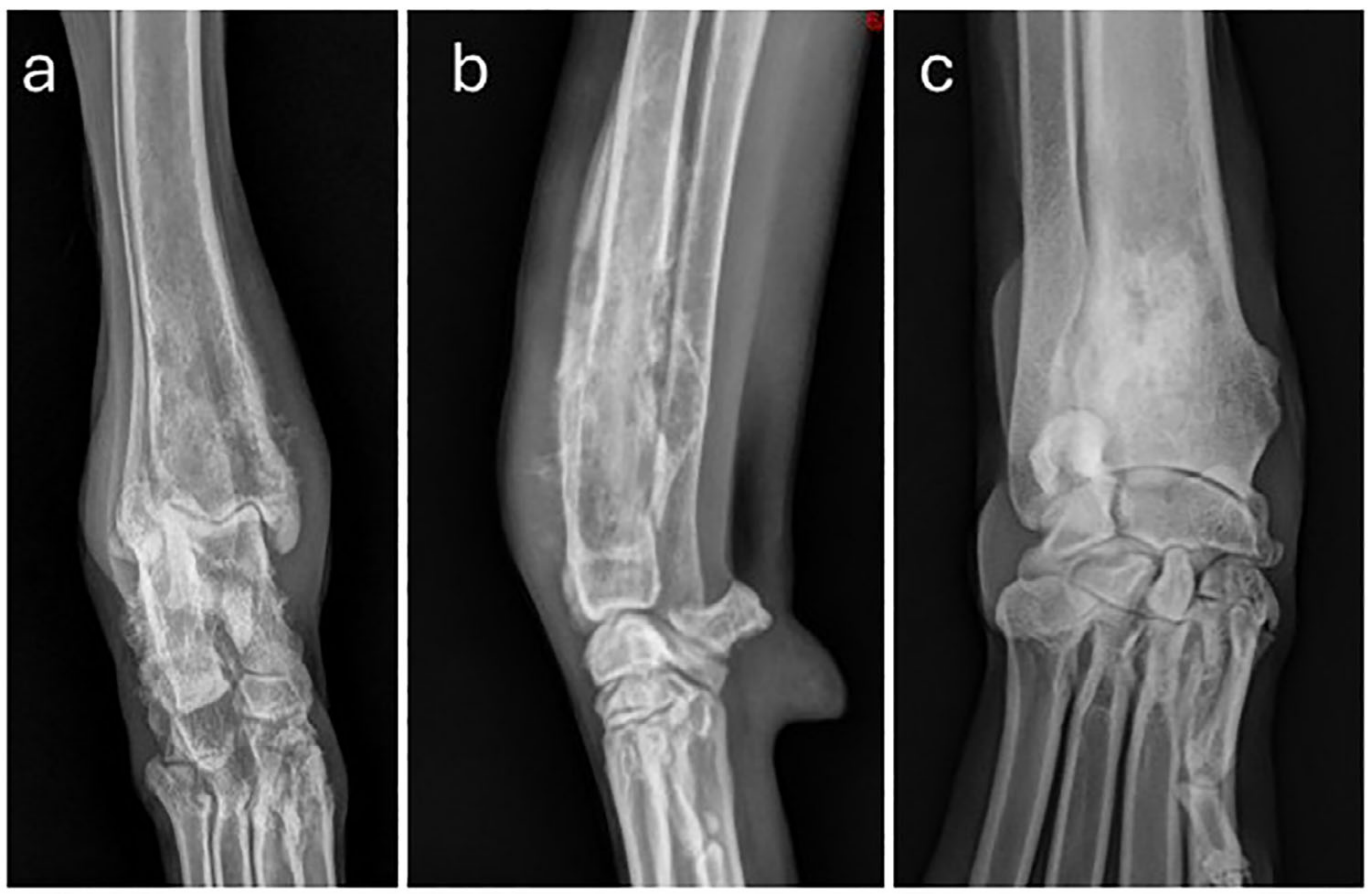
Representative radiographic images of lesions characterized as (a) primarily lytic (tibia metaphyseal and epiphyseal region, Patient 152), (b) mixed lytic and proliferative (radius mid diaphyseal to metaphyseal region, Patient 129), or (c) primarily proliferative (distal radius, Patient 196).

**FIGURE 9:**
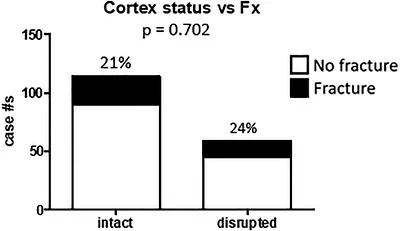
Comparison of intact or disrupted cortex on radiographic imaging versus incidence of pathologic fracture. Percentages listed per group represent percentages fractured, no statistically significant differences (*p* = 0.702, Fisher's exact test).

**FIGURE 10:**
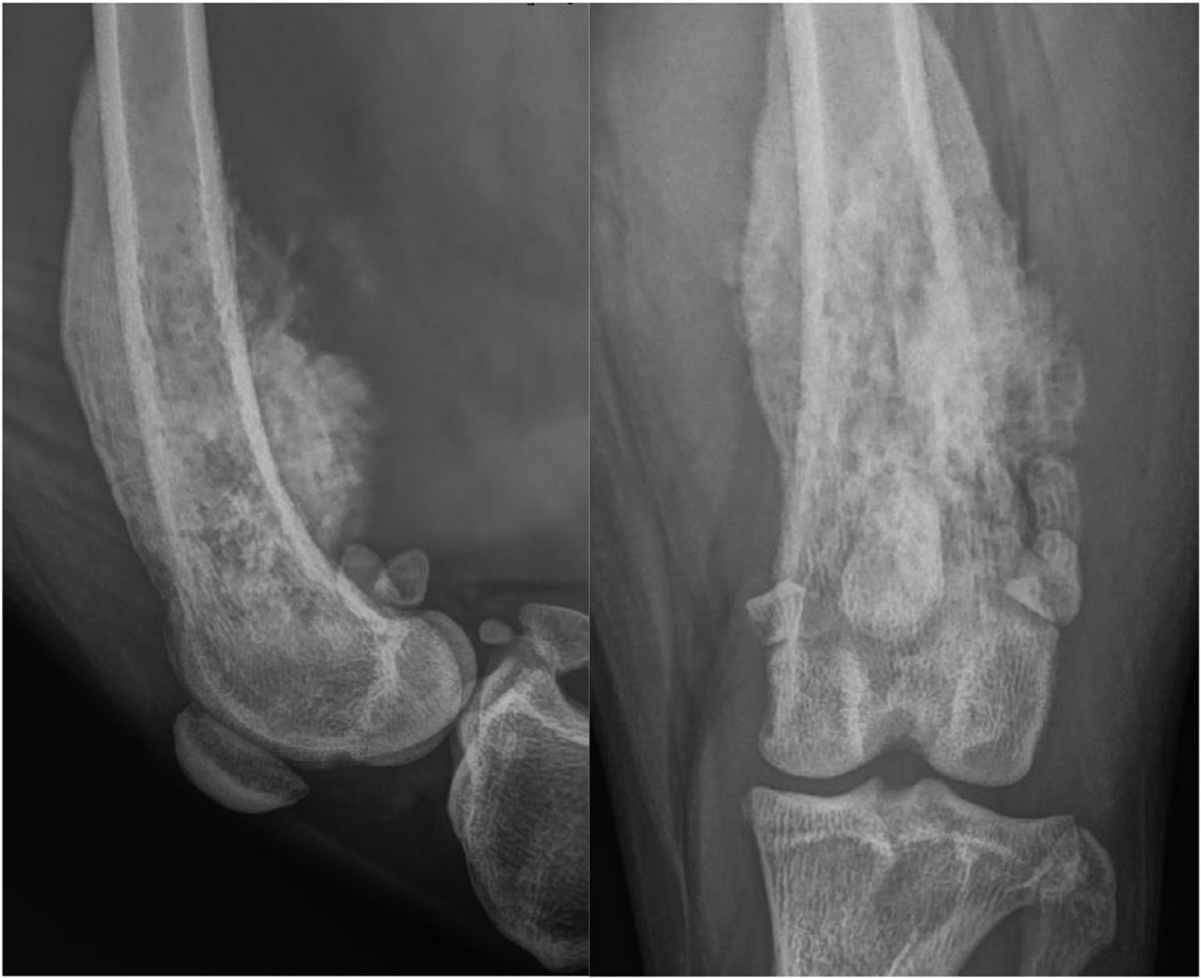
Representative radiographic images depicting cortical lysis (femur metaphyseal region, patient 178).

One patient initially presented with a very small pathologic fracture prior to radiation, which was not included as having a pathologic fracture in this study. Only three patients had a pathologic fracture that was suspected on the basis of physical exam or available records; remaining fractures were radiographically confirmed. One patient had a trauma‐related acute non‐weight‐bearing lameness without available radiographic imaging that led to the limb being amputated. Two dogs developed acute non‐weight‐bearing lameness that was assumed to be pathologic fractures by the primary care veterinarian, although radiographic confirmation was not available.

One patient had progressive pain and pulmonary metastasis but did not have an identified date of death. This dog's cause of death was presumed to be secondary to progressive pain based on medical record review. One patient had pathologic fracture of a metastatic rib mass and was not included as a pathologic fracture for analysis because this study was focused on the incidence of pathologic fractures at the irradiated site. One patient had pleural effusion and did not have an identified date of death. That case's cause of death was presumed to be secondary to pulmonary metastasis. Forty‐nine dogs were euthanized due to distant metastasis, 17 due to pathologic fracture, and 43 due to recurrent tumor or persistent intractable pain. Forty dogs were euthanized for non‐OSA‐related causes, 16 died, 16 were lost to follow‐up, and three were alive at the time of statistical analysis.

On average, patients were on 2.28 pain medications prior to RT and 2.9 medications post RT. Only five treatments led to a reduced number of pain medications being administered, so it was not statistically evaluated. No specific time point post RT was used, as the majority of patients did not have a reduction in pain medications administered.

Two patients in this study who were necropsied had confirmed spinal involvement of metastatic OSA. Four patients had progressive clinical signs associated with severe neurologic spinal dysfunction but were not identified definitively as secondary to OSA metastasis.

The median overall TTP for all events was 124 days. The median TTP for patients with distant metastasis as their first event was 158 days (*n* = 73). The median overall survival time for all patients was 243 days.

## 4 Discussion

In this study, none of the hypothesized risk factors were statistically linked to pathological fracture incidence. In addition, the observed rate of pathologic fracture with palliative radiation (21.7%) is lower than that reported in recent publications evaluating various coarsely fractionated protocols (35.7% and 30%) [12, 21].

We found no difference between SDR and LDR RT, despite the purported benefits of higher dose rates [22, 23]. The retrospective nature of the study may have obscured differences in efficacy or toxicity, but our results suggest that any putative temporal differences in cellular DNA repair mechanisms are not clinically significant. Steel et al. modeled cellular repair rates with prolonged delivery and concluded that increased delivery times result in loss of biologic effectiveness with increased time for cellular repair and survival [22, 23]. Late‐responding tissues have increased reparative responses when treated with dose rates below 1 Gy (100 cGy) per minute. Kann et al. highlighted the importance of considering source age, dose rate, and treatment time when formulating treatment protocols using Co‐60 [24]. Balamucki et al. [25] did not find any significant correlation between dose rate and reduced neuropathic pain relief using a Co‐60 source in humans. However, the lowest dose per fraction in Balamucki's study was significantly higher (80, 85, or 90 Gy in a single fraction) than is used in veterinary medicine for OSA palliation [25]. In our study, there were no statistically significant differences between the standard and LDR groups regarding rates of pathologic fracture or efficacy of treatment. It is possible that the duration of time over which radiation was delivered in the groups (2 min and up to 40 min, respectively) was not long enough to result in a significant difference, based on the cellular repair half‐life of underlying bone [22, 23].

No statistically significant differences in fracture rates were found on the basis of body weight or after comparing forelimb (55%–60% of weight‐bearing) and hind limb tumors. A small subset of dogs weighing less than 20 kg had no reported pathologic fractures, which could be related to decreased loading of bone. The almost ubiquitous use of zoledronate in this case series, pharmacologically maintaining bone density in the context of disease progression, could preempt any effect of skeletal loading on fracture incidence. Limb‐guarding to avoid loading‐related limb pain may have acted as an effective countermeasure against fracture despite differences in skeletal integrity and body weight.

The lack of association between radiological lesion categories or cortical bone continuity and eventual pathologic fracture was unexpected. In one recent retrospective study, dogs with predominantly lytic lesions were 3.22 times more likely to eventually fracture than proliferative or mixed tumors when only pain management was administered [26]. Clinicians often develop subjective opinions on the radiographic integrity of bone in and around the tumor. This can influence whether amputation versus palliative radiation is offered as first‐line therapy for local disease and pain relief. We found that predominantly lytic lesions or lesions with cortical defects are not predictive of subsequent pathological fracture. It is possible that the tumor‐associated matrix provides sufficient support for load‐bearing, in addition to post‐radiation fibrosis of the remaining stroma. When evaluating cortical bone loss, Observer 1 evaluated for at least one discontinuous cortex, whereas Observer 2 included cortical thinning without complete loss. This is a potential explanation for the disagreement rate regarding the presence of cortical bone loss. In light of these findings, the practice of recommending amputation on the basis of radiological appearance of the OSA lesion should be reconsidered.

A major difficulty in evaluating pathologic fracture incidence is that there is no clear consensus on what defines a pathologic fracture clinically. In the human literature, the definition of a pathologic fracture generally involves damage to a bone at a location of abnormal bone pathology. Despite this, it is still unclear how a pathologic fracture is defined. Some argue a pathologic fracture must be atraumatic or can involve some trauma [27, 28]. Osteoporotic fractures are variably excluded in epidemiologic studies as a cause of pathologic fracture [28]. Additionally, there is still overall difficulty in identifying stress fractures from pathologic fractures in humans, based on radiographic imaging characteristics [29]. In dogs, a sudden worsening in lameness associated with radiographic signs of bone displacement or cortical discontinuity is considered diagnostic, although terminal radiographs are, understandably, unavailable in some cases that worsen acutely. Comparisons of pathologic fractures between people and dogs are confounded by biomechanical differences between quadrupeds and bipeds, given that the number of propulsive legs at different gaits and speeds can cause changes in both body axis positioning and force distribution among limbs [30].

The use of systemic chemotherapy in dogs treated with palliative radiation for appendicular OSA has yielded inconsistent results. The behavior of OSA following amputation has been well characterized [1]. In contrast, patterns of metastasis and causes of morbidity and mortality are likely different when the leg is retained and treated with RT, consequently altering the benefits of chemotherapy. Significantly longer survival times have been found in some [31, 32, 33, 34], but not other [35, 36], retrospective case series that addressed the value of chemotherapy following palliative radiation. The reason(s) for the increased survival in previous studies, when reported, are unclear but could relate to delay of metastasis, variability in euthanasia decision‐making, or extended pain control. In people, decreased bone pain following platinum‐based chemotherapy has been reported [37]. In this case series, the improved survival following chemotherapy approached but did not reach statistical significance when evaluated by Mann–Whitney testing. It was, however, highly significant when evaluated by Gehan–Breslow–Wilcoxon testing. This test better accounts for the substantial number of patients in this cohort that died soon after their treatments. Because of inconsistent rechecks and lack of thoracic radiography at regular intervals, it is not possible to know whether this relates to local versus systemic tumor control.

The specific mechanism(s) responsible for pathologic fracture following RT are currently not well defined. It is possible that delayed radiation effects on late‐responding tissues, such as periosteum, bone, and blood vessels, interfere with the ability of peri‐lesional cell populations to respond effectively to the compromised skeletal integrity at the tumor location [38]. Further, increased limb‐loading that follows postradiation pain relief could also increase loading and contribute to the likelihood of pathological fracture.

Limitations of this study can be attributed to its retrospective nature. In the absence of medical records, surveying owners months or years after a dog's death risks recall bias regarding end‐of‐life decisions, timing, and diagnostic testing. Although most patients had definitive diagnostic testing consistent with OSA, this was not required given the characteristic findings with radiographic imaging, cytology, and clinical presentation. Additionally, there is inherent subjectivity when evaluating radiographs for cortical bone lysis, as it may over‐ or underestimate bone density. CT‐based measurement of cortical bone loss would be preferable but was not available for this cohort. An additional limitation is that it is inherently difficult to distinguish between radiation‐induced pathologic fracture and a disease‐related fracture. We used a delay of at least 3 months between treatment and fracture to distinguish between these etiologies, given the accepted definition of adverse radiation events in late‐responding tissues such as bone.

## 5 Conclusion

We evaluated the influence of radiation dose‐rate, tumor location, and patient body weight following palliative irradiation for canine appendicular OSA on pathologic fracture incidence. Radiation dose rate did not influence fracture incidence. This study provides a basis for interpreting the outcomes of studies using different dose rates and radiation beams (high‐energy x‐rays vs. gamma rays). Radiographic characteristics of the OSA lesions were not associated with pathologic fracture incidence. This suggests that the apparent lytic status of OSA lesions at presentation should not be used to influence decisions regarding limb amputation. The use of adjuvant chemotherapy was associated with improved survival times in this patient population but did not impact pathologic fracture incidence.

On the basis of our findings, a prospective clinical trial evaluating palliative RT with bisphosphonates, with or without chemotherapy, would better answer whether the increased survival time was related to delayed metastatic behavior. A consensus definition of pathologic fracture would strengthen conclusions of future investigations of this clinical problem.

## Author Contributions

Conception and design: Andrew Kurtzman and Kim A. Selting. Acquisition of data: Andrew Kurtzman, Elizabeth Rappleyea, Kim A. Selting, Jayme Looper, and Audrey Billhymer. Analysis and interpretation of data: Andrew Kurtzman, Kim A. Selting, and Matt Stewart. Drafting the article: Andrew Kurtzman and Kim A. Selting. Revising article for intellectual content: Andrew Kurtzman, Kim A. Selting, Jayme Looper, and Matt Stewart. Final approval of the completed article: Andrew Kurtzman, Kim A. Selting, and Matt Stewart.

## Funding

The authors have nothing to report.

## Disclosure

Previously presented as an abstract at ACVR 2023.

## Ethics Statement

This investigation followed standard ethical guidelines regarding the collection and reporting of retrospective data analysis of animal patients.

## Conflicts of Interest

The authors declare no conflicts of interest.

## Data Availability

Data can be provided upon request.

## References

[1] N. P. Ehrhart , S. D. Ryan , and T. M. Fan , “Tumors of the Skeletal System,” in Small Animal Clinical Oncology, 5th ed., ed. S. J. Withrow and MacEwen (WB Saunders Co, 2013), 463–503.

[2] A. Barger , R. Graca , K. Bailey , et al., “Use of Alkaline Phosphatase Staining to Differentiate Canine Osteosarcoma From Other Vimentin‐Positive Tumors,” Veterinary Pathology 42, no. 2 (2005): 161–165, 10.1354/vp.42-2-161. 15753469

[3] R. S. Brodey and W. H. Riser , “Canine Osteosarcoma. A Clinicopathologic Study of 194 Cases,” Clinical Orthopaedics and Related Research 62 (1969): 54–64. 5251443

[4] C. L. Fitzpatrick , J. P. Farese , R. J. Milner , et al., “Intrinsic Radiosensitivity and Repair of Sublethal Radiation‐Induced Damage in Canine Osteosarcoma Cell Lines,” American Journal of Veterinary Research 69 (2008): 1197–1202, 10.2460/ajvr.69.9.1197. 18764694

[5] A. Coomer , J. Jarese , R. Milner , J. Liptak , N. Bacon , and D. Lurie , “Radiation Therapy for Canine Appendicular Osteosarcoma,” Veterinary and Comparative Oncology 7 (2009): 15–27, 10.1111/j.1476-5829.2008.00177.x. 19222827

[6] L. A. Vakaet and T. Boterberg , “Pain Control by Ionizing Radiation of Bone Metastasis,” International Journal of Developmental Biology 48 (2004): 599–606, 10.1387/ijdb.041817lv. 15349834

[7] R. Zajaczkowska , M. Kocot‐Kepska , W. Leppert , and J. Worldliczek , “Bone Pain in Cancer Patients: Mechanisms and Current Treatment,” International Journal of Molecular Sciences 20 (2019): 6047, 10.3390/ijms20236047. PMC6928918 31801267

[8] S. Shor , B. A. Fadl‐Alla , H. C. Pondenis , et al., “Expression of Nociceptive Ligands in Canine Osteosarcoma,” Journal of Veterinary Internal Medicine 29, no. 1 (2015): 268–275, 10.1111/jvim.12511. PMC4858053 25572473

[9] P. Muir and C. P. Ruaux‐Mason , “Microcrack Density and Length in the Proximal and Distal Metaphyses of the Humerus and Radius in Dogs,” American Journal of Veterinary Research 61, no. 1 (January 2000): 6–8, 10.2460/ajvr.2000.61.6. 10630769

[10] E. M. Green , W. M. Adams , and L. J. Forrest , “Four Fraction Palliative Radiotherapy for Osteosarcoma in 24 Dogs,” Journal of the American Animal Hospital Association 38 (2002): 445–451, 10.5326/0380445. 12220029

[11] H. M. Knapp‐Hoch , J. L. Fidel , R. K. Sellon , and P. R. Gavin , “An Expedited Palliative Radiation Protocol for Lytic or Proliferative Lesions of Appendicular Bone in Dogs,” Journal of the American Animal Hospital Association 45 (2009): 24–32, 10.5326/0450024. 19122061

[12] C. Pagano , B. Boudreaux , and K. Shiomitsu , “Safety and Toxicity of an Accelerated Coarsely Fractionated Radiation Protocol for Treatment of Appendicular Osteosarcoma in 14 Dogs: 10 Gy × 2 Fractions,” Veterinary Radiology & Ultrasound 57 (2016): 551–556, 10.1111/vru.12389. 27374864

[13] M. N. Mayer and C. K. Grier , “Palliative Radiation Therapy for Canine Osteosarcoma,” Canadian Veterinary Journal 47 (2006): 707–709. PMC1482437 16898117

[14] F. Mueller , V. Poirier , K. Melzer , D. Nitzl , M. Roos , and B. Kaser‐Hotz , “Palliative Radiotherapy With Electrons of Appendicular Osteosarcoma in 54 Dogs,” In Vivo 10 (2005): 713–716. 15999538

[15] J. Wang , H. Wang , and H. Qian , “Biological Effects of Radiation on Cancer Cells,” Military Medical Research 5 (2018): 20, 10.1186/s40779-018-0167-4. PMC6026344 29958545

[16] C. C. Ling , L. E. Gerweck , M. Zaider , and E. Yorke , “Dose‐Rate Effects in External Beam Radiotherapy Redux,” Radiotherapy and Oncology 95 (2010): 261–268, 10.1016/j.radonc.2010.03.014. 20363041

[17] Q. Yao , R. Zheng , G. Xie , et al., “Late‐Responding Normal Tissue Cells Benefit From High‐Precision Radiotherapy With Prolonged Fraction Delivery Times via Enhanced Autophagy,” Scientific Reports 5 (2015): 9119, 10.1038/srep09119. PMC4357857 25766900

[18] P. A. Jeggo , V. Geuting , and M. Lobrich , “The Role of Homologous Recombination in Radiation‐Induced Double‐Strand Break Repair,” Radiotherapy and Oncology 101 (2011): 7–12, 10.1016/j.radonc.2011.06.019. 21737170

[19] J. D. Bourland , “Radiation Oncology Physics,” in Clinical Radiation Oncology, 3rd ed., ed. L. L. Gunderson , J. E. Tepper , and J. A. Bogart (Saunders‐Elsevier, 2012), 95–152, 10.1016/B978-1-4377-1637-5.00006-7.

[20] T. R. Reynolds , “Stresses on the Limbs of Quadrupedal Primates,” American Journal of Physical Anthropology 67, no. 4 (1985): 351–362, 10.1002/ajpa.1330670407. 4061589

[21] M. W. Nolan , N. A. Green , E. M. DiVito , B. D. X. Lascelles , and S. M. Haney , “Impact of Radiation Dose and Pre‐Treatment Pain Levels on Survival in Dogs Undergoing Radiotherapy With or Without Chemotherapy for Presumed Extremity Osteosarcoma,” Veterinary and Comparative Oncology 18, no. 4 (2020): 538–547, 10.1111/vco.12576. 32048435

[22] G. G. Steel , J. D. Down , J. H. Peacock , and T. C. Stephens , “Dose‐Rate Effects and the Repair of Radiation Damage,” Radiotherapy and Oncology 5, no. 4 (1986): 321–331, 10.1016/s0167-8140(86)80181-5. 3726169

[23] G. G. Steel , J. M. Deacon , G. M. Duchesne , A. Horwich , L. R. Kelland , and J. H. Peacock , “The Dose‐Rate Effect in Human Tumour Cells,” Radiotherapy and Oncology 9, no. 4 (1987): 299–310, 10.1016/s0167-8140(87)80151-2. 3317524

[24] B. H. Kann , J. B. Yu , J. M. Stahl , et al., “The Impact of Cobalt‐60 Source Age on Biologically Effective Dose in High‐Dose Functional Gamma Knife Radiosurgery,” Journal of Neurosurgery 125 (2016): 154–159, 10.3171/2016.6.GKS161497. 27903196

[25] C. J. Balamucki , V. W. Stieber , T. L. Ellis , et al., “Does Dose Rate Affect Efficacy? The Outcomes of 256 Gamma Knife Surgery Procedures for Trigeminal Neuralgia and Other Types of Facial Pain as They Relate to the Half‐Life of Cobalt,” Journal of Neurosurgery 105 (2006): 730–735, 10.3171/jns.2006.105.5.730. 17121135

[26] J. A. Rubin , J. N. Suran , D. C. Brown , and K. A. Agnello , “Factors Associated With Pathological Fractures in Dogs With Appendicular Primary Bone Neoplasia: 84 Cases (2007–2013),” Journal of the American Veterinary Medical Association 247, no. 8 (2015): 917–923, 10.2460/javma.247.8.917. 26421404

[27] C. P. Adler , “Pathologische Knochenfrakturen: Definition und Klassifikation,” Langenbecks Archiv fur Chirurgie Supplement II, Verhandlungen der Deutschen Gesellschaft fur Chirurgie Deutsche Gesellschaft fur Chirurgie Kongress (1989): 479–486. 2577585

[28] J. R. Curtis , A. J. Taylor , R. S. Matthews , et al., “"Pathologic" Fractures: Should These be Included in Epidemiologic Studies of Osteoporotic Fractures?,” Osteoporosis International: A Journal Established as Result of Cooperation Between the European Foundation for Osteoporosis and the National Osteoporosis Foundation of the USA 20, no. 11 (2009): 1969–1972. 10.1007/s00198-009-0840-2 PMC2766025 19184268

[29] L. M. Fayad , I. R. Kamel , S. Kawamoto , D. A. Bluemke , F. J. Frassica , and E. K. Fishman , “Distinguishing Stress Fractures From Pathologic Fractures: A Multimodality Approach,” Skeletal Radiology 34, no. 5 (2005): 245–259, 10.1007/s00256-004-0872-9. 15838703

[30] T. Weihmann , “The Smooth Transition From Many‐Legged to Bipedal Locomotion—Gradual Leg Force Reduction and Its Impact on Total Ground Reaction Forces, Body Dynamics and Gait Transitions,” Frontiers in Bioengineering and Biotechnology 9 (2022): 769684, 10.3389/fbioe.2021.769684. PMC8855104 35186911

[31] M. L. Oblak , S. E. Boston , G. Higginson , S. G. Patten , G. J. Monteith , and J. P. Woods , “The Impact of Pamidronate and Chemotherapy on Survival Times in Dogs With Appendicular Primary Bone Tumors Treated With Palliative Radiation Therapy,” Veterinary Surgery 41, no. 3 (April 2012): 430–435, 10.1111/j.1532-950X.2012.00968.x. 23253085

[32] M. A. Griffin , T. W. Martin , D. H. Thamm , and D. R. Worley , “Partial Ulnar Ostectomy, Stereotactic Body Radiation Therapy, and Palliative Radiation Therapy as Local Limb Sparing Treatment Modalities for Ulnar Tumors in Dogs,” Frontiers in Veterinary Science 10 (2023): 1172139, 10.3389/fvets.2023.1172139. PMC10368977 37502712

[33] O. Ramirez 3rd , R. K. Dodge , R. L. Page , et al., “Palliative Radiotherapy of Appendicular Osteosarcoma in 95 Dogs,” Veterinary Radiology & Ultrasound 40, no. 5 (1999): 517–522, 10.1111/j.1740-8261.1999.tb00385.x. 10528848

[34] C. J. Norquest , C. A. Maitz , D. A. Keys , et al., “Fracture Rate and Time to Fracture in Dogs With Appendicular Osteosarcoma Receiving Finely Fractionated Compared to Coarsely Fractionated Radiation Therapy: A Single Institution Study,” Veterinary Medicine and Science 8, no. 3 (2022): 1013–1024, 10.1002/vms3.782. PMC9122396 35263506

[35] M. E. Duffy , C. L. Anderson , K. Choy , and J. L. Fidel , “Metronomic Administration of Lomustine Following Palliative Radiation Therapy for Appendicular Osteosarcoma in Dogs,” Canadian Veterinary Journal 59, no. 2 (2018): 136–142. PMC5764213 29386671

[36] B. Ringdahl‐Mayland , D. H. Thamm , and T. W. Martin , “Retrospective Evaluation of Outcome in Dogs With Appendicular Osteosarcoma Following Hypofractionated Palliative Radiation Therapy With or Without Bisphosphonates: 165 Cases (2010–2019),” Frontiers in Veterinary Science 9 (2022): 892297, 10.3389/fvets.2022.892297. PMC9128609 35619604

[37] Ș. Spînu , D. Sur , V. Creciun , D. Moșoiu , and D. Ciurescu , “Efficacy of Chemotherapy in Pain Control of Patients With Cancer at the Early Phase of Their Disease,” Healthcare (Basel) 13, no. 8 (2025): 931, 10.3390/healthcare13080931. PMC12027393 40281880

[38] R. Pacheco and H. Stock , “Effects of Radiation on Bone,” Current Osteoporosis Reports 11, no. 4 (2013): 299–304, 10.1007/s11914-013-0174-z. 24057133

